# 

**Published:** 1890-05-03

**Authors:** 


### NEW OFFICES IN THE STRAND.

The Hospital *is now published at* **140, Strand, W.C.** *It has been found desirable to establish* The Hospital *in this central and convenient locality, owing to the rapid extension of the business of this paper of late.*

### NOTICE TO CORRESPONDENTS.

All MS., Letters, Books for Review, and other matters intended for the Editor should be addressed The Editor, The Lodge, Porchester Square, London, W.

The Editor cannot undertake to return rejected M.S., even when accompanied by stamped directed envelope.

**Notice to Advertisers and Subscribers.**

All Advertisements, Orders for Copies of The Hospital, and Business Communications should be addressed to The Publisher, *not to the Editor*, The Hospital, 140, Strand, W.C.

**Bound Volumes of "The Hospital."**

Vol. VI., containing full page portraits of T.R.H. the Prince and Princess of Wales, and Miss Florence Nightingale, is obtainable at 4s., post free.

Orders will be received for Vol. VII., 4s. post free. Cases for binding may be had of the Publisher, at 1s. 6d. each.

**To Residents, Secretaries, and Matrons.**

The Editor will be glad to have the Regulations and each Report as issued by Asylums, Hospitals, Convalescent and Nursing Institutions, as well as those of other Charities, and an early copy in duplicate of all Appeals and Festival Notices, etc., addressed to *The Editor, The Lodge, Porchester Square, London, W.* Any superintendent, secretary, matron, or other chief official who regularly sends such information may be registered, on application to the Publisher, to receive free of cost a cover for binding The Hospital in half-yearly volumes.

### Scraps and Gleanings.

Carlisle will keep Hospital Sunday on May 11th.

Malvern Rural Hospital treated 84 patients last year.

Nottingham Eye Infirmary treated 277 in-patients last year.

Leicester Provident Dispensary received over 29,000 visits from sick members last year.

Chichester has increased its bill for alcohol, while all other hospitals are decreasing theirs.

The Mayor of Norwich opened the annual sale of the Norwich Sick Poor Repository on April 23rd.

The Duke of Edinburgh presided on Monday at the annual meeting of the Mission to Seamen, of which he has long been patron.

The annual report of the Suffolk Convalescent Home is satisfactory; funds are coming in for the proposed new women's wing.

The Duke of Cambridge will lay the foundation stone of the new buildings of the London Hospital on May 20th at two p.m.

A successful meeting to consider the rules of the Convalescent Home for Friendly Society Members was held at Durham on April 24th.

Princess Christian visited the bazaar at Windsor, in aid of the Young Women's Christian Association, and made several purchases.

Two successful entertainments have been given at Abergavenny by the Amateur Snowflake Minstrels, in aid of the Children's Hospital.

At the quarterly court of the Exeter Hospital a cheque for £167 was acknowledged for the Nurses' Home, the result of the late tableaux.

The Brussels Anti-Slavery Conference has adopted, at a full sitting, the proposals for the suppression of the slave trade by sea which had been elaborated in committee.

Mr. P. J. Kingston, L.R.C.P., M.R.C.S., has been appointed house-surgeon at the Lock Hospital and Asylum, Harrow Road, W., in succession to Mr. H. Carlos Barr, L.R.C.P., M.R.C.S., resigned.

The trustees of the Clergy Pensions Institution have just received a most munificent donation of £10,000 for the charity from Mrs. Turner, who has already made a gift of £20,000 in the dioceses of York and Liverpool.

The honorary distinction of Professor of Psychology at King's College, London, has been conferred by the council upon Dr. Ernest White, who since 1887 has been medical superintendent and resident physician of the City of London Lunatic Asylum at Stone, near Dartford.

The committee on colour-blindness met last week, and elected Lord Rayleigh chairman. Amongst those present were Sir George Stokes, M.P., President of the Royal Society; Mr. Brudenell Carter, Mr. Francis Galton, and Dr. Farquharson, M.P. The committee propose to meet weekly.

The *Dentists' Register*, just published, shows that there are 4,805 dentists in the United Kingdom. Of these 1,079 are licentiates in dental surgery, 3,700 are persons on their own declaration in *bona fide* practice as dentists, and 26 possess additional surgical qualifications. The foreign dentists registered number 13.

Miss Eliza Brisco has bequeathed £2,500 to the Hastings Convalescent Home, Beau Site; £2,000 to the East Sussex Hospital, Hastings; £1,000 each to the Children's Convalescent Home (St. Leonards-on-Sea), All Saints' Convalescent Home (Eastbourne), All Saints' Orphanage (Margaret Street, Cavendish Square), the Brompton Hospital for Consumption, and the British Home for Incurables (Clapton).

### The Students' Column.

### DURHAM UNIVERSITY.

The following gentlemen have passed the first examination for the degree of Bachelor in Medicine at Durham University:

ELEMENTARY ANATOMY AND PHYSIOLOGY, CHEMISTRY WITH CHEMICAL PHYSICS, AND BOTANY WITH MEDICAL BOTANY.

Braithwaite, John, College of Medicine, Newcastle-upon-Tyne.
Browne, Frederick Henry, College of Medicine, Newcastle-upon-Tyne.
Bryant, Charles Hilary, College of Medicine, Newcastle-upon-Tyne.
Gamlen, Harold Ernest, College of Medicine, Newcastle-upon-Tyne.
Harker, William Edmund, College of Medicine, Newcastle-upon-Tyne.
McCoull, Stanley, College of Medicine, Newcastle-upon-Tyne.
Morison, Henry Bannerman, College of Medicine, Newcastle-upon-Tyne.
Richardson, Alfred Yarker, College of Medicine, Newcastle-upon-Tyne.
Smurthwaite, Harry, College of Medicine, Newcastle-upon-Tyne.
Thompson, Arthur Edward, College of Medicine, Newcastle-upon-Tyne.
Watson, Ogden, College of Medicine, Newcastle-upon-Tyne.

ELEMENTARY ANATOMY AND PHYSIOLOGY.

Crichton, Harry, College of Medicine, Newcastle-upon-Tyne.
Jackson, Daniel Noel, College of Medicine, Newcastle-upon-Tyne.
Keenan, Thomas Francis, College of Medicine, Newcastle-upon-Tyne.
Lloyd-Williams, Rowland Venables, Coll. Medicine, Newcastle-upon-Tyne.
Norman, Edward Fitzgibbon John, College of Medicine, Newcastle-upon-Tyne.
Visser, Thomas Christoffel, College of Medicine, Newcastle-upon-Tyne.

CHEMISTRY WITH CHEMICAL PHYSICS AND BOTANY WITH MEDICAL BOTANY.

Best, Harry George, Middlesex Hospital.
Cameron, Allan Gordon Russell, St. Mary's Hospital.
Challice, John, London Hospital.
Dow, William Alexander, St. Bartholomew's Hospital.
Fullerton, Francis William, St. Thomas's Hospital.
Hanks, Charles, College of Medicine, Newcastle-upon-Tyne.
Jones, Frank Silva, London Hospital.
Morris, Bedlington Howel, College of Medicine, Newcastle-upon-Tyne.
Preston, William James Darbyshire, Coll. of Med., Newcastle-upon-Tyne.

CHEMISTRY WITH CHEMICAL PHYSICS.

Hutton, James Alfred, M.R.C.S., L.R.C.P., Middlesex Hospital.

